# Propofol via Antioxidant Property Attenuated Hypoxia-Mediated Mitochondrial Dynamic Imbalance and Malfunction in Primary Rat Hippocampal Neurons

**DOI:** 10.1155/2022/6298786

**Published:** 2022-01-18

**Authors:** Jingfeng Han, Weiping Tao, Wei Cui, Jiawei Chen

**Affiliations:** Department of Anesthesiology, Jing'an District Central Hospital, No. 259 Xi Kang Road, Shanghai 200040, China

## Abstract

**Background:**

Hypoxia may induce mitochondrial abnormality, which is associated with a variety of clinical phenotypes in the central nervous system. Propofol is an anesthetic agent with neuroprotective property. We examined whether and how propofol protected hypoxia-induced mitochondrial abnormality in neurons.

**Methods:**

Primary rat hippocampal neurons were exposed to propofol followed by hypoxia treatment. Neuron viability, mitochondrial morphology, mitochondrial permeability transition pore (mPTP) opening, mitochondrial membrane potential (MMP), and adenosine triphosphate (ATP) production were measured. Mechanisms including reactive oxygen species (ROS), extracellular regulated protein kinase (ERK), protein kinase A (PKA), HIF-1*α*, Drp1, Fis1, Mfn1, Mfn2, and Opa1 were investigated.

**Results:**

Hypoxia increased intracellular ROS production and induced mPTP opening, while reducing ATP production, MMP values, and neuron viability. Hypoxia impaired mitochondrial dynamic balance by increasing mitochondrial fragmentation. Further, hypoxia induced the translocation of HIF-1*α* and increased the expression of Drp1, while having no effect on Fis1 expression. In addition, hypoxia induced the phosphorylation of ERK and Drp1^ser616^, while reducing the phosphorylation of PKA and Drp1^ser637^. Importantly, we demonstrated all these effects were attenuated by pretreatment of neurons with 50 *μ*M propofol, antioxidant *α*-tocopherol, and ROS scavenger ebselen. Besides, hypoxia, propofol, *α*-tocopherol, or ebselen had no effect on the expression of Mfn1, Mfn2, and Opa1.

**Conclusions:**

In rat hippocampal neurons, hypoxia induced oxidative stress, caused mitochondrial dynamic imbalance and malfunction, and reduced neuron viability. Propofol protected mitochondrial abnormality and neuron viability via antioxidant property, and the molecular mechanisms involved HIF-1*α*-mediated Drp1 expression and ERK/PKA-mediated Drp1 phosphorylation.

## 1. Introduction

Mitochondria are semiautonomous and double-membrane organelles that provide the most proportion of energy for living cells through citric acid cycle and oxidative phosphorylation. They are dynamic organelles, and their morphology reflects the balance between fusion and fission process. Fusion merges the contents of adjoined mitochondria, creating a homogenous network of elongated mitochondria, while fission is necessary during cell division. Correct regulation of mitochondrial fusion/fission equilibrium is essential for mitochondrial function, cellular homoeostasis, and functions [1]. Proper mitochondrial dynamics and function are especially important for neurons due to their enrichment of mitochondria and extremely high energetic demands, with maintaining resting membrane potentials and firing of action potentials [2]. Therefore, it is no doubt that mitochondrial abnormality inevitably leads to neural dysfunction and neurological disorders [3].

It has been recognized that neurons are vulnerable to multiple harmful stimuli, such as hypoxia [4], which serves as a major pathologic risk factor for Parkinson's disease [5], Alzheimer's disease [6], and cerebral ischemic stroke [7]. Speaking of the underlying mechanisms, accumulating evidence indicates that oxidative stress and resultant mitochondrial abnormality are important contributing factors and potential mechanisms for hypoxia-related neuron injury [8–10] and neurodegenerative diseases [11].

Propofol (2, 6-diisopropyl phenol) is a widely used intravenous anesthetic agent during general anesthesia and sedation. Apart from anesthetic advantages, it has been reported to possess antioxidative effects [12] and neuroprotective properties [13]. On cellular level, it was shown propofol may protect mouse hippocampal neurons and microglia from hypoxia- and oxidative stress-induced apoptosis [14, 15]. In addition, propofol may protect hypoxia-impaired integrity of blood-brain barrier (BBB) in the *in vitro* model [16, 17]. A recent study from our group indicated that propofol may reverse hypoxia- and TNF-*α*-mediated dysregulation of brain-derived neurotrophic factor (BDNF)/tropomyosin receptor tyrosine kinase B (TrkB) pathway and may protect neuron viability in primary rat hippocampal neurons [18]. Considering the pivotal role of oxidative stress in mitochondrial abnormality and the antioxidant property of propofol, the effect of propofol on mitochondrial abnormality is of great interests. Previously, propofol infusion syndrome has been reported in genetically confirmed mitochondrial disorder patients [19], and accordingly, propofol was considered as a mitochondrion-toxic agent [20]. In contrast, a recent animal study found only high dosage over a prolonged duration of propofol administration is mitochondrion-toxic and may lead to mitochondrial disorders, and the mechanism may involve disrupted electron flow along respiratory chain and disrupted mitochondria permeability transition pore (mPTP) and mitochondrial membrane potential (MMP) [21]. And it was revealed that short-term application of propofol showed no harm to mitochondria and should be safe even in mitochondrial disorder models [21]. Importantly, a recent study indicated propofol may reduce oxidative stress and protect mitochondria in hypoxia-treated cardiac cells [22]. Also, an *in vitro* study carried out in hippocampal neurons demonstrated propofol could alleviate ischemia-reperfusion- (I/R-) induced MMP loss and thus protect hippocampal neurons from I/R injury [23].

In this *in vitro* study, we aimed to reveal whether propofol could modulate hypoxia-mediated mitochondrial abnormality such as dynamic imbalance and malfunction in primary rat hippocampal neurons and further investigated the potential underlying mechanisms. Neuron viability, mitochondrial morphology, mPTP opening, MMP, and adenosine triphosphate (ATP) production were used as parameters for evaluating mitochondrial abnormality.

## 2. Materials and Methods

### 2.1. Experimental Design

Primary rat hippocampal neurons were cultured in normoxic condition (95% air and 5% CO_2_) till ready for experiments. To establish hypoxic situation, neurons were maintained in a chamber flushed with a gas mixture (90% N_2_, 5% O_2_, and 5% CO_2_) for different duration (0, 1, 2, 3, 6, and 12 h). To investigate the effect of propofol, neurons were incubated with different concentrations (1, 5, 10, 25, 50, and 100 *μ*M) of propofol for 1 h followed by exposure to hypoxia treatment. We intended to identify the effect of propofol on hypoxia-induced mitochondrial abnormality, such as mitochondrial dynamic imbalance and malfunction. More importantly, we aimed to investigate the underlying mechanisms, including reactive oxygen species (ROS), extracellular regulated protein kinases (ERK), protein kinase A (PKA), hypoxia-inducible factor-1*α* (HIF-1*α*), Drp1, Fis1, Mfn1, Mfn2, and Opa1. To confirm the pivotal role of these factors, specific inhibitors and activators were applied.

### 2.2. Cell Culture

Primary rat hippocampal neurons were purchased from ScienCell Research Laboratories (Carlsbad, CA, USA). The cryopreserved neurons were thawed and seeded into tissue culture flasks containing 5 ml Neurobasal™-A culture medium (Gibco, Grand Island, NY, USA) supplemented with neuronal growth supplement, 5% fetal bovine serum (FBS), and 1% penicillin/streptomycin, and kept in a humidified incubator filled with 5% CO_2_ and 95% humidified air at 37°C. The culture media were replaced every 2-3 days, until neurons reached approximately 70% confluency and were ready for experiments without subculturing.

### 2.3. Hypoxia Treatment and Propofol Pretreatment Protocol

To examine the effect of hypoxia on hippocampal neurons, once the neurons were ready for experiments, the culture flasks were transferred to a humidified hypoxia-controlled incubator chamber flushed with a gas mixture (90% N_2_, 5% O_2_, and 5% CO_2_) at 37°C for different duration (0, 1, 2, 3, 6, and 12 h). We aimed to determine the time point, at which hypoxia exerted an effect on mitochondrial dynamic balance and function.

To investigate the potential protective effect of propofol in hypoxia-treated hippocampal neurons, neurons were incubated with different concentrations (1, 5, 10, 25, 50, and 100 *μ*M) of propofol (Sigma-Aldrich, St Louis, MO, USA) or its solvent 0.1% dimethyl sulfoxide (DMSO, Sigma-Aldrich, St Louis, MO, USA) for 1 h followed by exposure to hypoxia treatment. By observing mitochondrial dynamic balance and function, we intended to figure out the optimal concentration, at which propofol exerted a protective effect against hypoxia-induced mitochondrial dynamic imbalance and malfunction.

### 2.4. Cell Viability Assay

In this study, hippocampal neuron viability was assessed by 3-(4,5-Dimethylthiazol-2-yl)-2,5-diphenyltetrazolium bromide (MTT) assay as described previously [22]. MTT is a dye that could interact with succinate dehydrogenase in the mitochondria of living cells, forming insoluble formazan crystals. Accordingly, the MTT assay was especially sensitive to mitochondria abnormality-related neuron injury. Briefly, neurons were seeded in 6-well culture plates and exposed to respective treatment. Then, culture media were removed, and neurons were rinsed with phosphate-buffered saline (PBS). MTT was added to the neurons at a final concentration of 0.5 mg/ml and incubated for 4 h at 37°C. After forming purple formazan crystals, 0.1% DMSO was added to dissolve them for approximately 1 h in the dark. A microplate reader (Bio-Rad, Hercules, California, USA) was used to determine the absorbance values at 490 nm. Neuron viability was expressed as the percentage of absorbance of treated neurons compared with that of untreated control neurons, which was set as 100%.

### 2.5. Mitochondrial Morphology Evaluation

Mitochondrial morphology was evaluated by measuring mitochondrial fragmentation count through confocal laser scanning fluorescence microscopy with laser scanning confocal microscope (TCS SP5, Leica Microsystems Inc., Buffalo Grove, IL, USA). In brief, after being seeded on sheet glasses and subjected to treatment, neurons were fixed in 4% paraformaldehyde for 15 min, permeabilized with Triton-X 100 (0.1%) for 15 min, and then incubated with 100 nM Mito-Tracker Red (Invitrogen, Carlsbad, CA, USA) for 15 min at room temperature to stain the mitochondrial membrane. Finally, neurons were imaged at 600× magnification and analyzed with Image J software. Mitochondrial fragmentation count was evidenced by the organelles' number. Data were expressed as ratio of mitochondrial fragmentation count of treated neurons compared with that of untreated control neurons, which was set as 100%.

### 2.6. Mitochondrial Membrane Potential (MMP) Determination

MMP was determined with the use of MMP indicator fluorescence dye rhodamine-123 (R123) with the use of fluorescence-activated cell sorter (FACS). Briefly, after treatment, neurons were washed and incubated with R123 solution (Beyotime Institute of Biotechnology, Shanghai, China) at a final concentration of 1 *μ*M at 37°C in a dark chamber for 30 min. Then, neurons were washed with PBS to remove excess dye, and BD FACSPresto™ System (BD Biosciences, San Jose, CA, USA) was used to detect the fluorescent signal of R123 at an excitation wavelength of 490 nm and an emission wavelength of 535 nm. Data were expressed as a percentage of mean ± standard deviation of fluorescent intensity of R123 staining of treated neurons compared with that of untreated control neurons, which was set as 100%.

### 2.7. Mitochondrial Permeability Transition Pore (mPTP) Opening Assessment

The mPTP opening was assessed by using MitoProbe™ Transition Pore Assay Kit (Thermo Fisher Scientific, Waltham, MA, USA) according to the manufacturer's instruction. Briefly, after treatment, culture media were removed and neurons were incubated with 1 *μ*M calcein-AM (Life Technologies, Grand Island, NY, USA) at 37°C in the dark. Then, 1 mM CoCl_2_ was added and incubated with neurons for another 15 min. The fluorescence of neurons was measured with BD FACSPresto™ System (BD Biosciences, San Jose, CA, USA), and data were expressed as a percentage of mean ± standard deviation of calcein fluorescence absorbance unit (A.U) of treated neurons compared with that of untreated control neurons, which was set as 100%.

### 2.8. Intracellular Adenosine Triphosphate (ATP) Evaluation

Intracellular ATP level was determined by a commercialized assay kit (Beyotime, Shanghai, China) according to the manufacturer's instructions. In brief, neurons were incubated in 6-well plates and subject to respective treatment. Then, the neurons were cooled to room temperature for 10 min, suspended in solution containing 0.22 M sucrose, 0.12 M mannitol, 40 mM Tricine, and 1 mM EDTA (pH 7.5) and subject to ClarioStar plate reader (BMG Labtech, Ortenberg, Germany). Due to a linear relation between emitted light and ATP content, intracellular ATP was calculated via linear regression of a standard curve. The data were as recorded as relative luminescence unit divided by the amount of protein (RLU/mg protein) and expressed as percentage of mean ± standard deviation of treated neurons compared with that of untreated control neurons, which was set as 100%.

### 2.9. Intracellular ROS Measurement

Intracellular ROS was monitored by ROS-sensitive fluorescent probe which could penetrate into neurons rapidly and was hydrolyzed by ROS into green fluorescent. In brief, after treatment, neurons were incubated with 10 *μ*M fluorescent probe 2,7-dichlorofluorescin diacetate (DCFH-DA, Beyotime Institute of Biotechnology, Shanghai, China) for 20 min at 37°C. Afterward, neurons were washed with PBS and subject to fluorescence microplate reader with an excitation wavelength of 488 nm and an emission wavelength of 525 nm. The data were recorded as a percentage of mean ± standard deviation of fluorescence intensity in treated neurons compared with that of untreated neurons, which was set as 100%.

### 2.10. Intracellular Superoxide Anion Measurement

Superoxide anion generation in hippocampal neurons was measured with a superoxide anion-sensitive chemiluminescent probe coelenterazine. In brief, after treatment, neurons were washed, harvested, and suspended in Krebs-Ringer buffer containing 10 *μ*M coelenterazine for 30 min. The chemiluminescence of coelenterazine was detected on a scintillation counter (LS 7000, Beckman Coulter Inc., Brea, CA, USA) with a single active photomultiplier tube. Data were recorded as a percentage of mean ± standard deviation of chemiluminescence in treated neurons compared with that of untreated control neurons, which was set as 100%.

### 2.11. Preparation of Whole Cell Extracts

After respective treatment, hippocampal neurons were washed with PBS and scraped off the culture flasks. After centrifugation for 5 min at 1000 revolutions per minute (rpm), neurons were suspended in RIPA cell lysis buffer (Santa Cruz Biotechnology, Santa Cruz, CA, USA) containing 1% protease inhibitor and 0.1% phosphatase inhibitor for 5 min. Total cellular proteins were obtained by centrifuging for 5 min at 3000 rpm and quantified by BCA assay kit (Beyotime Institute of Biotechnology, Shanghai, China).

### 2.12. Preparation of Nuclear Extracts

Nuclear extracts were prepared with the use of Nuclear Extract Kit (Active Motif, Carlsbad, California, USA) according to the manufacturer's instructions. After removing culture media, neurons were washed with PBS and scraped off, transferred to a prechilled tube, pelleted by centrifugation at 500 rpm for 5 min at 4°C, suspended in a hypotonic buffer, and incubated on ice for 15 min. After adding detergent and vortexing for 10 sec, the suspensions were centrifuged at 14,000 rpm for 1 min at 4°C. The pellets were then suspended in complete lysis buffer, vortexed for 10 sec, and incubated for 30 min at 4°C. The suspensions were centrifuged at 14,000 rpm for 10 min at 4°C, and the supernatant was collected. Protein concentration of the supernatant was quantified by BCA assay kit (Beyotime Institute of Biotechnology, Shanghai, China).

### 2.13. Protein Analysis by Western Blot Analysis

Equal amounts of protein (about 60 *μ*g) were separated through SDS-PAGE and electrophoretically transferred to polyvinylidinene fluoride membranes. Nonspecific binding was eliminated by incubation with 5% skimmed milk at room temperature for 2 h, and specific antibodies (Cell Signaling Technology, Beverly, MA, USA) against ERK, phosphorylated ERK, PKA, phosphorylated PKA, HIF-1*α*, Drp1, phosphorylated Drp1 at ser 616 (p-Drp1^ser616^), phosphorylated Drp1 at ser 637 (p-Drp1^ser637^), Fis1, Mfn1, Mfn2, Opa1, or GAPDH were then incubated with the membranes for overnight at 4°C. Subsequently, the membranes were washed with TBST and incubated with corresponding HRP-conjugated secondary antibody (Santa Cruz Biotechnology, Santa Cruz, CA, USA) at room temperature for 2 h. Protein bands were visualized with Amersham ECL plus Western blotting detection reagent (Santa Cruz Biotechnology, Santa Cruz, CA, USA), and images were recorded with Odyssey System (LI-COR Biosciences, Lincoln, NE, USA) and semiquantified with Image J v1.8.0 software.

### 2.14. Statistical Analysis

Data were presented as mean ± standard deviation. All experiments were conducted with five independent repeats, which were performed with different cultures. Differences between groups were assessed with paired, two-tailed Student's *t*-test or one-way ANOVA, followed by post hoc Tukey testing. All statistical analyses were performed with SPSS software 11.5, and a significant difference was set at *p* < 0.05.

## 3. Results

### 3.1. Hypoxia Induced Mitochondrial Dynamic Imbalance and Malfunction in a Time-Related Manner

**Figure 1:**
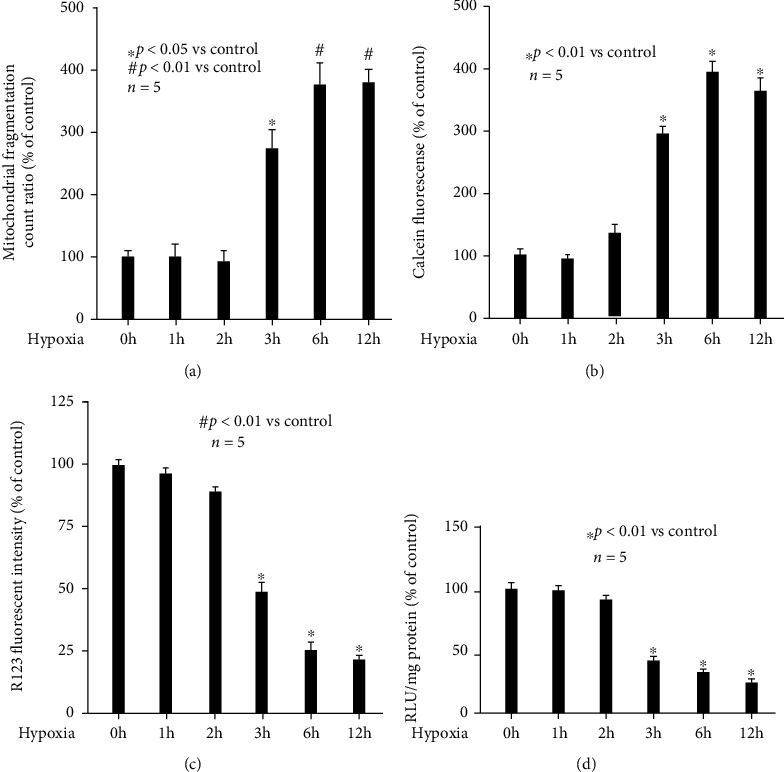
Hypoxia induced mitochondrial abnormality. Hypoxia treatment for 0 h was considered as normoxic condition and served as control. Data were presented as the percentage of mean ± standard deviation of treated neurons compared with that of untreated control neurons, which was set as 100%, and *n* represents the number of independent repeats. (a) Hypoxia impaired mitochondrial dynamic balance in a time-related manner. (b) Hypoxia induced mPTP opening in a time-related manner. (c) Hypoxia reduced MMP values in a time-related manner. (d) Hypoxia reduced intracellular ATP content in a time-related manner.

To evaluate the effect of hypoxia on mitochondrial dynamics and function, rat hippocampal neurons were exposed to hypoxia treatment (90% N_2_, 5% O_2_, and 5% CO_2_) for different duration (0, 1, 2, 3, 6, and 12 h). Mitochondrial dynamics was assessed by observing mitochondrial morphology (mitochondrial fragmentation), while mitochondrial function was evaluated by measuring mPTP opening, MMP value, and ATP production. We observed that hypoxia impaired mitochondrial dynamic balance, favoring fission process, in a time-related manner (Figure 1(a)), and our data indicated that mitochondrial fragmentation count increased after 3 h hypoxia treatment (*p* < 0.05 vs. control) and reached peak level after 6 h hypoxia treatment (*p* < 0.01 vs. control). In addition, we revealed that hypoxia induced mPTP opening (Figure 1(b)), while reduced MMP values (Figure 1(c)) and ATP content (Figure 1(d)) in a time-related manner. Precisely, these hypoxia-induced effects appeared after 3 h hypoxia treatment (*p* < 0.01 vs. control) and peaked after 6-12 h hypoxia treatment (*p* < 0.01 vs. control). Based on above data, hypoxia treatment (90% N_2_, 5% O_2_, and 5% CO_2_) for 3 h was considered as an optimal treatment condition to induce mitochondrial abnormality in rat hippocampal neurons.

### 3.2. Hypoxia-Mediated Effects on Mitochondrial Dynamic Imbalance and Malfunction Were Alleviated by Propofol Pretreatment

**Figure 2:**
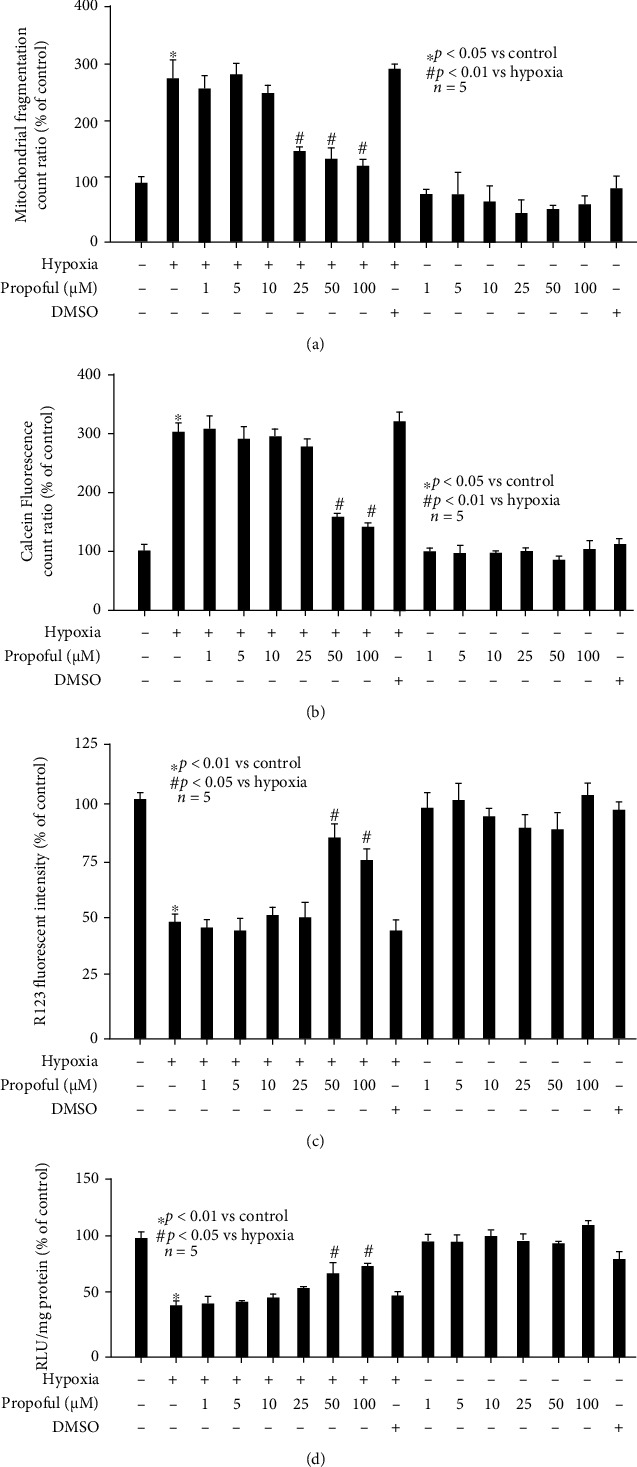
Hypoxia-mediated effects on mitochondrial abnormality were alleviated by propofol pretreatment. Hypoxia treatment for 0 h was considered as normoxic condition and served as control. “-” represents the absence of specific treatment, and “+” represents the presence of specific treatment. Data were presented as the percentage of mean ± standard deviation of treated neurons compared with that of untreated control neurons, which was set as 100%, and *n* represents the number of independent repeats. (a) 25, 50, and 100 *μ*M propofol reversed hypoxia-mediated mitochondrial dynamic imbalance. (b) 50 and 100 *μ*M propofol reversed hypoxia-mediated mPTP opening. (c) 50 and 100 *μ*M propofol reversed hypoxia-mediated MMP values. (d) 50 and 100 *μ*M propofol reversed hypoxia-mediated intracellular ATP content.

To investigate whether hypoxia-mediated detrimental effects on mitochondrial dynamics and function were affected by propofol, hippocampal neurons were pretreated with different concentrations (1, 5, 10, 25, 50, and 100 *μ*M) of propofol or its solvent 1% DMSO for 1 h, followed by hypoxia treatment (90% N_2_, 5% O_2_, and 5% CO_2_, 3 h). We detected that 25, 50, and 100 *μ*M propofol reversed hypoxia-mediated mitochondrial dynamic imbalance (*p* < 0.01 vs. hypoxia, Figure 2(a)), while 50 and 100 *μ*M propofol reversed hypoxia-mediated mPTP opening (*p* < 0.01 vs. hypoxia, Figure 2(b)), MMP values (*p* < 0.05 vs. hypoxia, Figure 2(c)), and ATP content (*p* < 0.05 vs. hypoxia, Figure 2(d)). Further, our data indicated that hypoxia-mediated effects were not affected by DMSO (Figure 2). Interestingly, we revealed propofol alone had no effect on mitochondrial function (Figures 2(a)–2(d)), but 50 and 100 *μ*M propofol slightly (not significantly) reduced mitochondrial fragmentation (Figure 2(a)). Based on these findings, we inferred the protective property of propofol against hypoxia-mediated detrimental effects was independent of DMSO, and therefore, focused on the mechanism responsible for the beneficial property of 50 *μ*M propofol.

### 3.3. Hypoxia-Induced Intracellular ROS Accumulation Was Mitigated by Propofol, *α*-Tocopherol, or Ebselen Pretreatment

**Figure 3:**
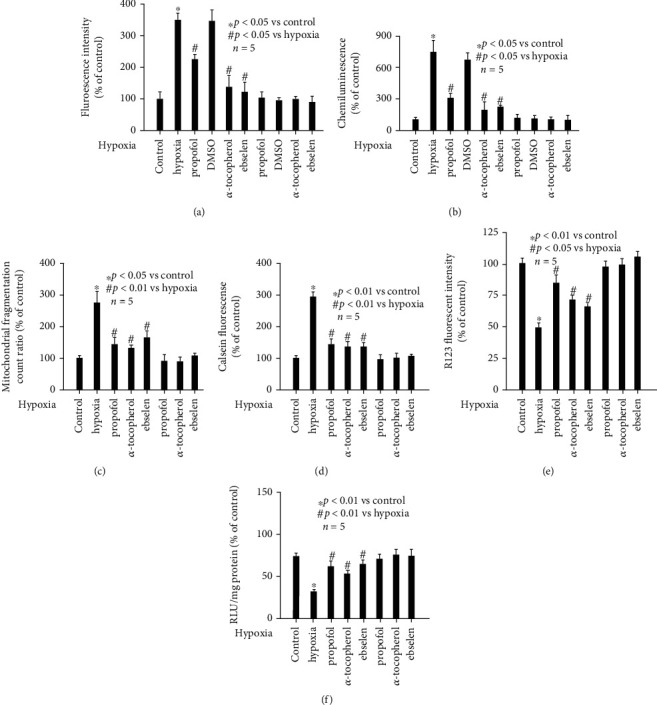
Hypoxia-induced intracellular oxidative stress and mitochondrial abnormality was mitigated by propofol, *α*-tocopherol, or ebselen. Data were presented as the percentage of mean ± standard deviation of treated neurons compared with that of untreated control neurons, which was set as 100%, and *n* represents the number of independent repeats. (a) Hypoxia induced intracellular ROS accumulation, which was attenuated by propofol, *α*-tocopherol, and ebselen. (b) Hypoxia induced intracellular superoxide anion generation, which was attenuated by propofol, *α*-tocopherol, and ebselen. (c) Hypoxia-induced effect on mitochondrial dynamic balance was attenuated by propofol, *α*-tocopherol, and ebselen. (d) Hypoxia-induced effect on mPTP opening was attenuated by propofol, *α*-tocopherol, and ebselen. (e) Hypoxia-induced effect on MMP value was attenuated by propofol, *α*-tocopherol, and ebselen. (f) Hypoxia-induced effect on intracellular ATP content was attenuated by propofol, *α*-tocopherol, and ebselen.

Since hypoxia has been proved to induce ROS in multiple cell types, and ROS has been proved to control cellular function by regulating mitochondria homeostasis, we aimed to examine the effects of hypoxia and propofol on intracellular ROS accumulation and the role of ROS in mitochondrial homeostasis. We pretreated neurons with 50 *μ*M propofol, 0.1% DMSO, 0.1 mM *α*-tocopherol (a potent antioxidant), or 40 *μ*M ebselen (a specific ROS scavenger) followed by hypoxia treatment (90% N_2_, 5% O_2_, and 5% CO_2_, 3 h). Compared with untreated neurons, hypoxia induced intracellular ROS accumulation (*p* < 0.05 vs. control, Figure 3(a)), which was attenuated by 50 *μ*M propofol, 0.1 mM *α*-tocopherol, and 40 *μ*M ebselen (*p* < 0.05 vs. hypoxia, Figure 3(a)), rather than 0.1% DMSO. To confirm this finding, we measured intracellular superoxide anion (a specific ROS) and demonstrated that hypoxia induced intracellular superoxide anion level (*p* < 0.01 vs. control, Figure 3(b)), which was also attenuated by 50 *μ*M propofol, 0.1 mM *α*-tocopherol, and 40 *μ*M ebselen (*p* < 0.01 vs. hypoxia, Figure 3(b)), but not by 0.1% DMSO. Thereafter, we solidly believed the effect of propofol was not due to DMSO.

### 3.4. Hypoxia-Induced Mitochondrial Dynamic Imbalance and Malfunction Was Mitigated by *α*-Tocopherol or Ebselen Pretreatment

In addition, we revealed that hypoxia-induced effects (mitochondrial morphology, mPTP opening, MMP value, and ATP production) in hippocampal neurons were all attenuated by 0.1 mM *α*-tocopherol and 40 *μ*M ebselen pretreatment (Figures 3(c)–3(f)), and the extent was comparable to that of propofol.

### 3.5. Hypoxia-Impaired Neuron Viability Was Attenuated by Propofol, *α*-Tocopherol, or Ebselen Pretreatment

**Figure 4:**
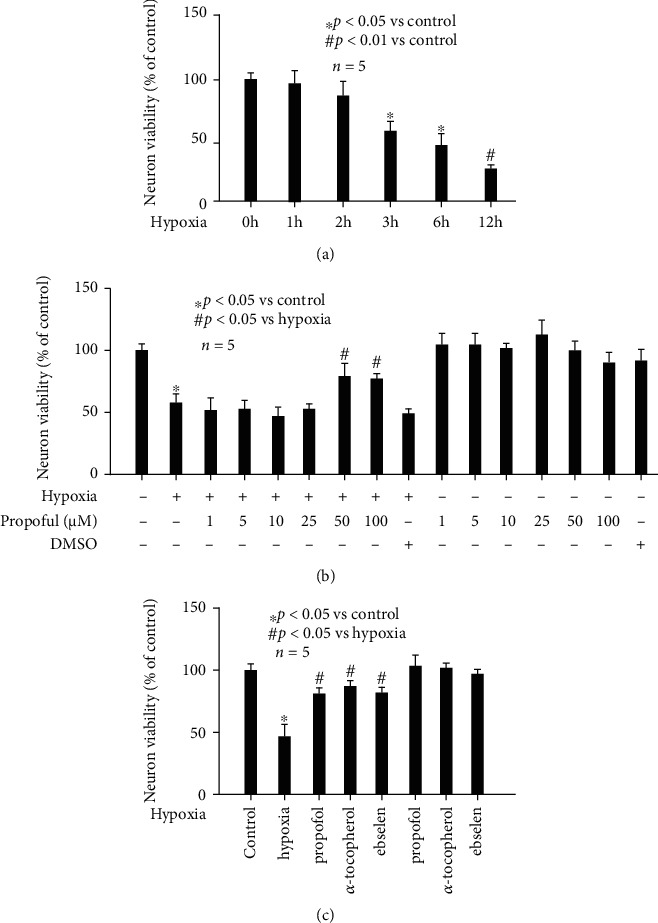
Hypoxia-impaired neuron viability was attenuated by propofol, *α*-tocopherol, or ebselen pretreatment. Hypoxia treatment for 0 h was considered as normoxic condition and served as control. Data were presented as the percentage of mean ± standard deviation of treated neurons compared with that of untreated control neurons, which was set as 100%, and *n* represents the number of independent repeats. (a) Hypoxia reduced neuron viability in a time-related manner. (a) 50 and 100 *μ*M propofol attenuated hypoxia-impaired neuron viability. (c) Hypoxia-impaired neuron viability was attenuated by *α*-tocopherol and ebselen.

When primary rat hippocampal neurons were ready for experiments, they were exposed to hypoxia treatment (90% N_2_, 5% O_2_, and 5% CO_2_) for different duration (0, 1, 2, 3, 6, and 12 h), and neuron viability was determined by MTT assay. As shown in Figure 4(a), we found hypoxia reduced neuron viability in a time-related manner, and the inhibitory effects appeared after 3 h treatment (*p* < 0.05 vs. control) and peaked after 12 h treatment (*p* < 0.01 vs. control). To investigate whether hypoxia-mediated detrimental effects on neuron viability was affected by propofol, hippocampal neurons were pretreated with different concentrations (1, 5, 10, 25, 50, and 100 *μ*M) of propofol or its solvent 1% DMSO for 1 h, followed by hypoxia treatment (90% N_2_, 5% O_2_, and 5% CO_2_, 3 h). As shown in Figure 4(b), 50 and 100 *μ*M propofol attenuated hypoxia-impaired neuron viability (*p* < 0.05 vs. hypoxia). Further, we revealed that hypoxia-mediated effects on neuron viability were also attenuated by 0.1 mM *α*-tocopherol and 40 *μ*M ebselen pretreatment (Figure 4(c)), and the extent was comparable to that of propofol.

### 3.6. Propofol Modulated Hypoxia-Mediated Expression and Phosphorylation of Mitochondrial Fission-Related Molecule Drp1

**Figure 5:**
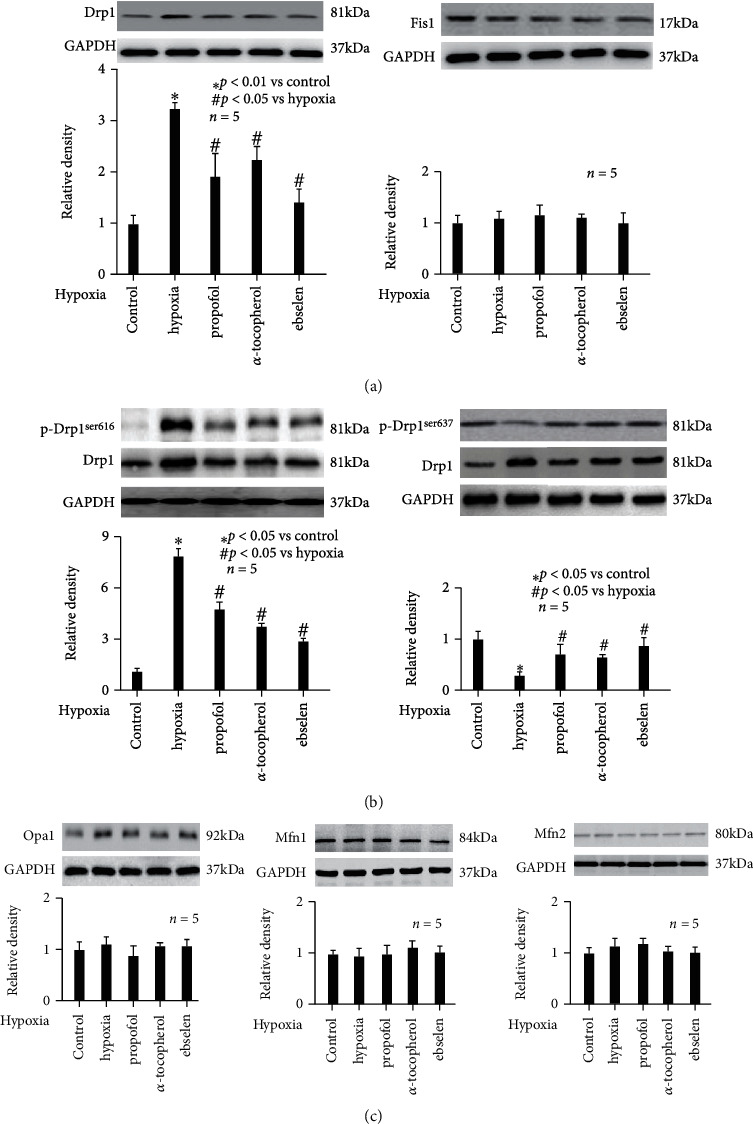
Propofol alleviated hypoxia-mediated expression and phosphorylation of Drp1. The upper panel was a representative experiment, and the lower panel was the summary of densitometric data from 5 separate experiments. GAPDH served as loading control. (a) Hypoxia induced the protein expression of Drp1 (left) but not Fis1 (right), and hypoxia-mediated Drp1 expression was attenuated by 50 *μ*M propofol, 0.1 mM *α*-tocopherol, and 40 *μ*M ebselen. Data were expressed as normalized ratio of protein band density of Drp1 or Fis1 against GAPDH and were presented as mean ± standard deviation. (b) Hypoxia induced the phosphorylation of Drp1 at ser 616 (p-Drp1^ser616^), while reduced the phosphorylated of Drp1 at ser 637 (p-Drp1^ser637^), which were both attenuated by propofol, *α*-tocopherol, and ebselen. Data were expressed as normalized ratio of protein band density of phosphorylated Drp1 against Drp1, which was normalized with GAPDH, and were presented as mean ± standard deviation. (c) Hypoxia, propofol, *α*-tocopherol, and ebselen had no effect on the expression of Opa1 (left), Mfn1 (middle) or Mfn2 (right). Data were expressed as normalized ratio of protein band density of Opa1, Mfn1, or Mfn2 against GAPDH, and were presented as mean ± standard deviation.

To investigate the mechanism underlying hypoxia- and propofol-mediated mitochondrial morphology change, which depends on dynamic balance between fission and fusion process, we first examined the protein expression and phosphorylation status of mitochondrial fission-related protein Drp-1 and Fis1. As shown in Figure 5(a), we showed hypoxia treatment (90% N_2_, 5% O_2_, and 5% CO_2_, 3 h) induced the protein expression of Drp1 (left, *p* < 0.01 vs. control), while had no effect on Fis1 expression (right). In addition, hypoxia induced the phosphorylation of Drp1 at ser 616 (p-Drp1^ser616^) (*p* < 0.05 vs. control, Figure 5(b) left), while reduced the phosphorylated of Drp1 at ser 637 (p-Drp1^ser637^) (*p* < 0.05 vs. control, Figure 5(b) right). More importantly, we found hypoxia-mediated expression and phosphorylation of Drp1 were attenuated by pretreatment of neurons with 50 *μ*M propofol, 0.1 mM *α*-tocopherol, and 40 *μ*M ebselen (*p* < 0.05 vs. hypoxia, Figures 5(a) and 5(b)). It was noted that propofol had no effect on Fis1 expression (Figure 5(a) right).

In addition, we investigated mitochondrial fusion-related protein Opa1, Mfn1, and Mfn2. Our data indicated hypoxia had no effect on their expression, which was also not affected by 50 *μ*M propofol, 0.1 mM *α*-tocopherol, or 40 *μ*M ebselen (Figure 5(c)).

### 3.7. HIF-1*α*, ERK, and PKA Were Involved in Hypoxia- and Propofol-Modulated Expression and Phosphorylation of Drp1

**Figure 6:**
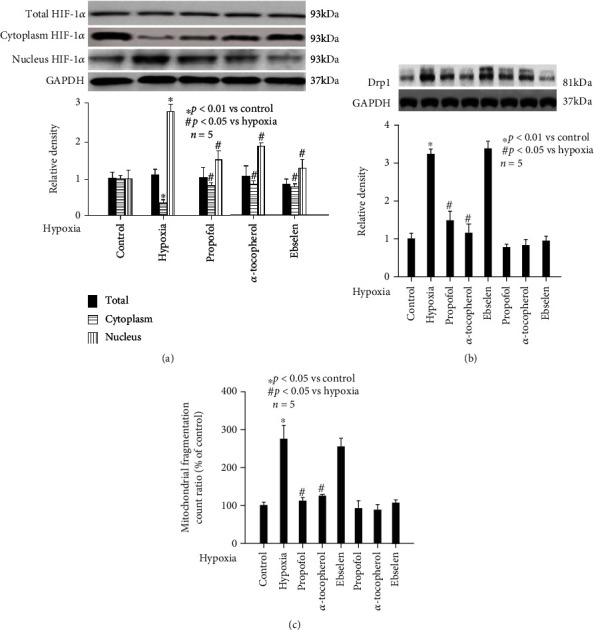
Hypoxia- and propofol-modulated Drp1 expression and mitochondrial dynamics were mediated through HIF-1*α*. The upper panel was a representative experiment, and the lower panel was the summary of densitometric data from 5 separate experiments. GAPDH served as loading control. Data were expressed as normalized ratio of protein band density of target protein against GAPDH and were presented as mean ± standard deviation. (a) Hypoxia-induced translocation of HIF-1*α* from cytoplasm to nucleus was abolished by propofol, *α*-tocopherol, and ebselen. (b) Hypoxia-induced Drp1 expression was mitigated by HIF-1*α* inhibitors. (c) Hypoxia-mediated mitochondrial fragmentation was attenuated by HIF-1*α* inhibitors. Data were presented as the percentage of mean ± standard deviation of treated neurons compared with that of untreated control neurons, which was set as 100%, and *n* represents the number of independent repeats.

The findings of the above experiments indicated that among all mitochondrial fission- and fusion-related proteins, only the modulation of Drp1 was consistent with that of mitochondrial morphology. As such, we investigated the potential underlying signaling pathway. As shown in Figure 6(a), we revealed that hypoxia treatment (90% N_2_, 5% O_2_, and 5% CO_2_, 3 h) induced the translocation of HIF-1*α* from the cytoplasm to nucleus (*p* < 0.01 vs. control), and this was abolished by pretreatment of neurons with 50 *μ*M propofol, 0.1 mM *α*-tocopherol, or 40 *μ*M ebselen (*p* < 0.05 vs. hypoxia, Figure 6(a)). Our data also revealed that hypoxia-induced Drp1 expression was mitigated by pretreatment of neurons with 10 *μ*M echinomycin (HIF-1*α* inhibitor) or 10 *μ*M oltipraz (HIF-1*α* inhibitor), rather than their solvent DMSO (*p* < 0.05 vs. hypoxia, Figure 6(b)). Consistently, we showed that hypoxia-mediated mitochondrial morphology change was attenuated by 10 *μ*M echinomycin and 10 *μ*M oltipraz (*p* < 0.05 vs. hypoxia, Figure 6(c)).

**Figure 7:**
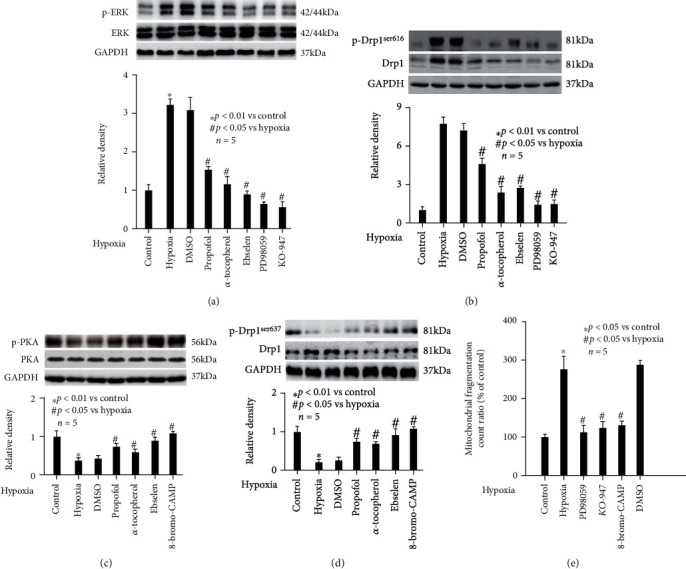
Hypoxia- and propofol-modulated phosphorylation of Drp1 and mitochondrial dynamics was mediated via ERK and PKA pathway. The upper panel was a representative experiment, and the lower panel was the summary of densitometric data from 5 separate experiments. GAPDH served as loading control. (a) Hypoxia induced the phosphorylation of ERK, which was abolished by propofol, *α*-tocopherol, ebselen, and ERK inhibitors. Data were expressed as normalized ratio of protein band density of phosphorylated ERK against ERK, which was normalized with GAPDH, and were presented as mean ± standard deviation. (b) Hypoxia induced the phosphorylation of Drp1^ser616^, which was abolished by propofol, *α*-tocopherol, ebselen, and ERK inhibitors. Data were expressed as normalized ratio of protein band density of phosphorylated Drp1 against Drp1, which was normalized with GAPDH, and were presented as mean ± standard deviation. (c) Hypoxia reduced the phosphorylation of PKA, which was abolished by propofol, *α*-tocopherol, ebselen, and PKA activator. Data were expressed as normalized ratio of protein band density of phosphorylated PKA against PKA, which was normalized with GAPDH, and were presented as mean ± standard deviation. (d) Hypoxia reduced the phosphorylation of Drp1^ser637^, which was abolished by propofol, *α*-tocopherol, ebselen, and PKA activator. Data were expressed as normalized ratio of protein band density of phosphorylated Drp1 against Drp1, which was normalized with GAPDH, and were presented as mean ± standard deviation. (e) Hypoxia-mediated mitochondrial fragmentation was attenuated by ERK inhibitors and PKA activator. Data were presented as the percentage of mean ± standard deviation of treated neurons compared with that of untreated control neurons, which was set as 100%, and *n* represents the number of independent repeats.

Meanwhile, we found that hypoxia treatment induced the phosphorylation of ERK (*p* < 0.01 vs. control, Figure 7(a)) and Drp1^ser616^ (*p* < 0.05 vs. control, Figure 7(b)), while reducing the phosphorylation of PKA (*p* < 0.01 vs. control, Figure 7(c)) and Drp1^ser637^ (*p* < 0.05 vs control, Figure 7(d)). And all these effects were abolished by pretreatment of neurons with 50 *μ*M propofol, 0.1 mM *α*-tocopherol, or 40 *μ*M ebselen (*p* < 0.05 vs. hypoxia, Figures 7(a)–7(d)). Importantly, we showed hypoxia-mediated phosphorylation of ERK and Drp1^ser616^ was attenuated by pretreatment of neurons with 10 *μ*M PD98059 (a selective ERK inhibitor) or 10 *μ*M KO-947 (a potent ERK inhibitor) (*p* < 0.05 vs. hypoxia, Figures 7(a) and 7(b)) rather than their solvent DMSO. Also, hypoxia-mediated phosphorylation of PKA and Drp1^ser637^ was attenuated by pretreatment of neurons with 10 *μ*M 8-Bromo-cAMP (PKA activator) rather than its solvent DMSO (*p* < 0.05 vs. hypoxia, Figures 7(c) and 7(d)). Consistently, we showed hypoxia-mediated mitochondrial morphology change was attenuated by 10 *μ*M PD98059, 10 *μ*M KO-947, and 10 *μ*M 8-Bromo-cAMP, but not by DMSO (*p* < 0.05 vs. hypoxia, Figure 7(e)).

## 4. Discussion

In this study, we demonstrated propofol may protect hypoxia-induced mitochondrial dynamic imbalance and malfunction in primary rat hippocampal neurons. Further, our data strongly implied that the protection of propofol against mitochondrial malfunction was carried out through its antioxidant property. In addition, we suggested that the protection against mitochondrial dynamic imbalance was mediated via modulating the expression and phosphorylation Drp1, and more importantly, we indicated the underlying mechanism may involve ROS, HIF1*α*, ERK, and PKA.

### 4.1. The Detrimental Effects and Underlying Mechanisms of Hypoxia-Mediated Mitochondrial Abnormality in Hippocampal Neurons

Hippocampus is a region of the brain fundamental for learning and memory. Numerous evidences indicated that structural, morphological, and electrophysiological alterations of hippocampus may be induced by pathophysiological insults such as ischemia/hypoxia [24], and altered structure and function of hippocampus were correlated with neurological disorders such as dementia [24], Alzheimer's disease [25], and postoperative cognitive dysfunction [26]. However, the exact underlying molecular mechanism is far from clear. Since hippocampal neurons are the most abundant component of hippocampus and are rich in mitochondria, which are also vulnerable to ischemia/hypoxia injury [27], and we inferred mitochondrial abnormality at least participates in hypoxia-induced hippocampal neuron and hippocampus malfunction.

It is now clear that mitochondrial defects are associated with a large variety of clinical phenotypes. This is the result of the mitochondria's central role in energy production, reactive oxygen species homeostasis, and cell death. Generally, mitochondrial abnormality refers to their morphological and functional alterations. Mitochondria exist in a dynamic network comprised of individual organelles that continuously join (fusion) and fragment (fission), and mitochondrial morphology reflects the balance of fusion and fission. Mitochondrial function is usually assessed by mPTP opening, MMP value, and ATP production.

In the current study, we investigated the effect and mechanism of hypoxia on mitochondrial function. As shown in Figure 1, we proved that hypoxia impaired mitochondrial function in rat hippocampal neurons. Furthermore, our data suggested intracellular ROS played a critical role, since both antioxidant and ROS scavenger reduced ROS (Figure 3) and abolished hypoxia-induced mitochondrial malfunction (Figure 3). The pivotal role of ROS during hypoxia-induced mitochondrial malfunction was also supported by previous publications, which demonstrated that hypoxia may impair mitochondrial function through inducing oxidative stress in rat cardiomyocytes [28], human renal tubular cells [29], murine fibroblasts [30], rat cerebral neuronal glial cells [31], and primary rat hippocampal neurons [32].

On the other hand, we also focused on the effect and mechanism of hypoxia-modulated mitochondrial morphology, which represents their dynamic balance. We found hypoxia increased mitochondrial fragmentation count (Figure 1(a)), which means hypoxia broke the balance between mitochondrial fusion and fission. Although we did not investigate the fusion and fission process separately, our data about mitochondrial fragmentation count implied hypoxia impaired mitochondrial dynamics, favoring mitochondrial fission process (Figure 1(a)). Consistently, we showed that hypoxia increased mitochondrial fission-related protein Drp1 expression (Figure 5(a)), while having no effect on mitochondrial fusion-related protein Opa1, Mfn1, and Mfn2 (Figure 5(c)). As such, we hypothesized that hypoxia impaired mitochondrial dynamics mainly through inducing fission process and left fusion process untouched. Drp1 is a member of the dynamin family of large GTPases and is recruited from the cytosolic compartment to mitochondria by adaptor proteins, including the outer mitochondrial transmembrane protein Fis1. Although Fis1 was also reported to play a role in mitochondrial fission [33], we found it was not affected by hypoxia (Figure 5(a)). In addition, we reported in hippocampal neurons hypoxia-induced Drp1 expression was due to hypoxia-sensitive transcription factor HIF-1*α* (Figure 6(a)), which was consistent with previous publications carried out in fibroblasts [30], cardiac cells [22], and vascular smooth muscle cells [34]. Although other transcription factors such as Smad2/3 [35], NF-*κ*B [36], and calcium-related transcription factors [37] have been reported to correlate with Drp1 expression, we demonstrated that HIF-1*α* inhibitors could almost completely inhibit hypoxia-induced Drp1 expression (Figure 6(b)), and thus, suggesting the pivotal role of HIF-1*α*.

Besides expression level, Drp1 activity is modulated by posttranslational modifications including phosphorylation, ubiquitination, sumoylation, and s-nitrosylation. Drp1 phosphorylation plays a crucial role in Drp1 activity regulation [38], and Drp1 may be modified by different kinases at different sites. It was believed that in hippocampal neurons, phosphorylation of Drp1 at serine 616 (p-Drp1^ser 616^) is positively correlated with mitochondrial fission while phosphorylation of Drp1 at serine 637 (p-Drp1^ser 637^) is negatively correlated with mitochondrial fission [38]. We, together with other groups [39], proved hypoxia significantly increased p-Drp1^ser 616^ (Figure 7(b)), while decreased p-Drp1^ser 637^ (Figure 7(d)). In addition, we testified that p-Drp1^ser 616^ was mediated through ERK (Figure 7(a)), while p-Drp1^ser 637^ was mediated through PKA (Figure 7(c)). Although other kinases, such as JNK [35], calcium-calmodulin kinase (CaMK) II [40], and PTEN-induced putative kinase 1 [41], were reported to be involved in Drp1 phosphorylation in neurons, our study with the use of specific inhibitor/activator strongly implied the critical role of ERK and PKA.

### 4.2. The Beneficial Property and Mechanisms of Propofol against Hypoxia-Mediated Mitochondrial Abnormality in Hippocampal Neurons

Ischemia/hypoxia is a well-known risk factor during surgical procedures and may cause injuries in multiple organs and tissues, especially in the central nervous system. As such, how to deal with ischemia/hypoxia-mediated detrimental effects in the brain perioperatively is a worldwide issue troubling anesthesiologists. Propofol is a widely used sedative agent during clinical anesthesia and has been reported to protect ischemia/hypoxia-mediated damages in neurons [42], microglia [15], in the *in vitro* blood-brain barrier [16], and in the animal model of rat brains [43]. Although the underlying mechanisms responsible for neuroprotective effects of propofol are extensively investigated, no consensus is reached. Recently, the potential role of oxidative stress-related signaling pathway is highly accepted [44, 45]. The antioxidant property of propofol has been extensively studied, and there are three major mechanisms concerning how propofol exerts antioxidant effects. First, propofol may directly scavenge free radicals and peroxynitrite due to its shared phenol structure with *α*-tocopherol. Second, propofol may suppress the biosynthesis and activity of nitric oxidase and NADPH oxidase, thus, reducing the production of oxidative stress. Third, propofol may induce the expression of antioxidant enzyme heme oxygenase-1 and superoxide dismutase, thus, facilitating the removal of oxidative stress. Although, in the current study we did not examine the detailed mechanism, our findings that propofol showed comparable effects to *α*-tocopherol, implying the antioxidant property of propofol in hippocampal neurons is due to oxidative stress scavenging effects. In addition, on cellular level, propofol was reported to prevent ROS and apoptosis by regulating iron homeostasis in human neuroblastoma cells [46], to reduce ROS and block calcium signaling pathway, thus, restoring synaptic protein expression in hippocampal neurons [47], and to attenuate ROS and ROS-induced toxicity in rat astroglial cells [48]. Since mitochondria are highly sensitive to oxidative stress, the effect of propofol on ROS-mediated mitochondria abnormality is of great interest. It was reported propofol may improve the ischemia-reperfusion injury-impaired structure and function of mitochondria in rat hippocampal neurons [49]. It was also reported that propofol protected cerebral ischemia-reperfusion via regulating mitochondrial depolarization [50]. In addition, propofol was demonstrated to inhibit mitochondrial fission evoked by oxygen-glucose deprivation in rat hippocampal neurons [51]. In the current study, we focused on the effect and mechanism of propofol on mitochondrial malfunction and dynamic imbalance, which were induced by hypoxia in hippocampal neurons. In consistence with previous publications carried out in cardiac cells [52], hepatic cells [53], and microglia [54], we proved propofol may protect hypoxia-induced mitochondrial malfunction and dynamic imbalance through its antioxidant property in hippocampal neurons (Figure 3).

It is well known that mitochondrial morphology reflects the balance between fusion and fission, which rely on mitochondrial fission-related proteins (Drp1 and Fis1) and mitochondrial fusion-related proteins (Opa1, Mfn1, and Mfn2). Drp1 is a member of the dynamin family of large GTPases and is considered as a critical mitochondrial fission-related protein. It is recruited from the cytosolic compartment to mitochondria and severs mitochondria by a GTP hydrolysis-dependent mechanism^53^. In addition to expression, Drp1's function results from phosphorylation by regulatory kinases. It was realized that phosphorylation of Drp1 at serine 616 and activates Drp1 and fission, and conversely, phosphorylation of Drp1 at Serine 637 inhibits its activity and fission [55]. Upon activation, Drp1 moves from the cytosol to the mitochondria where it assembles in multimers that constrict and divide the mitochondria. Our data implied the protective mechanism of propofol against mitochondrial dynamic imbalance mainly involved HIF-1*α*-modulated Drp1 expression and ERK-modulated Drp1 phosphorylation at serine 616 and PKA-modulated Drp1 phosphorylation at serine 637. We also checked Fis1 but found no modulation by hypoxia or propofol (Figure 5(a)). In addition, we inferred that hypoxia-induced hippocampal neuron injury was due to mitochondrial abnormality. This was supported by our findings that interventions by propofol may attenuate hypoxia-mediated mitochondrial abnormality and hippocampal neuron viability (Figure 4).

In the present in vitro study, the beneficial concentration of propofol was 50 *μ*M, which is about 9 *μ*g/ml. In clinical practice, during general anesthesia with propofol, its plasma concentration was usually kept at 2-6 *μ*g/ml. It seems 50 *μ*M is beyond physiological concentrations range and may not be achieved during general anesthesia. However, it is noted that in vitro system is different from *in vivo* system, and it is acknowledged the concentration of an agent used in vitro study maybe 10 times higher than that used in clinical practice and is still acceptable to explore mechanisms. Accordingly, we believe 50 *μ*M propofol in this study is clinically relevant.

### 4.3. Limitations

Although we believed the current project is a complete study and is of significant novelty, we realized the existence of several limitations. First, it is known that intracellular ROS was produced through multiple enzymes mainly composed of, but not limited to, NADPH oxidase, nitric oxidase, amine oxidase, oxalate oxidase, and peroxidases. Although it was reported that hypoxia may increase ROS production in microglia by inducing NADPH oxidase 2 [56], and it was shown that hypoxia induced ROS generation via NADPH oxidase 4 in mouse hippocampus [57], highly implying the role of NADPH oxidase, we did not investigate the exact pathway responsible for ROS production in the current study. Second, intracellular oxidative status depends on the balance between the generation and removal of ROS, which may involve superoxide dismutases, catalase, and glutathione peroxidase. However, we only measured the intracellular amount of ROS, without investigating its production and removal. Third, although our data indicated a correlation between mitochondrial abnormality and cell viability, we did not examine whether apoptosis, necrosis, or autophagy was responsible for impaired neuron viability.

## 5. Conclusions

In this study, we discovered in hippocampal neurons propofol exerted a beneficial effect against hypoxia-mediated neuron viability and mitochondrial abnormality and implied the mechanism may involve oxidative stress, HIF-1*α*-mediated Drp1 expression, and ERK/PKA-modulated Drp1 phosphorylation. Our findings raised a therapeutic rationale for choosing propofol as an anesthetic agent for those patients who are vulnerable to hypoxia injury during periopearative period.

## Data Availability

The data that support the findings of this study are available from the corresponding author upon reasonable request.
